# Robot-aided electrospinning toward intelligent biomedical engineering

**DOI:** 10.1186/s40638-017-0075-1

**Published:** 2017-11-10

**Authors:** Rong Tan, Xiong Yang, Yajing Shen

**Affiliations:** 1 City University of Hong Kong, Tat Chee Avenue, Kowloon, Hong Kong, SAR; 2 Centre for Robotics and Automation, CityU Shen Zhen Research Institute, Shen Zhen, China

**Keywords:** Robotics, Electrospinning, Biomedical engineering

## Abstract

The rapid development of robotics offers new opportunities for the traditional biofabrication in higher accuracy and controllability, which provides great potentials for the intelligent biomedical engineering. This paper reviews the state of the art of robotics in a widely used biomaterial fabrication process, i.e., electrospinning, including its working principle, main applications, challenges, and prospects. First, the principle and technique of electrospinning are introduced by categorizing it to melt electrospinning, solution electrospinning, and near-field electrospinning. Then, the applications of electrospinning in biomedical engineering are introduced briefly from the aspects of drug delivery, tissue engineering, and wound dressing. After that, we conclude the existing problems in traditional electrospinning such as low production, rough nanofibers, and uncontrolled morphology, and then discuss how those problems are addressed by robotics via four case studies. Lastly, the challenges and outlooks of robotics in electrospinning are discussed and prospected.

## Introduction

The basic idea of electrospinning originated in the period from 1934 to 1944, when researchers describes the use of electrostatic force to produce polymer filament device. The main principle is using high-voltage electrostatic field to stimulate the polymer charged jet and then to obtain the polymer nanofibers by charged jet curing. From the middle of the twentieth century to present, electrospinning technology ended up more than 60 years of silence, and finally in the last decade of twentieth century ushered in its glorious era. In 1994, “electrospinning” became a professional term, instead of “electrostatic spinning,” officially declared electrospinning as an independent academic field, began its research and development in the field of nanotechnology and related bioengineering. At present, electrospinning is rapidly emerging as a unique and versatile technique for the preparation of smooth nanofibers with controllable morphology from various polymers [1, 2]. The nanofibers produced by electrospinning have high surface area and highly porous structure, and furthermore, design flexibility is an important advantage of electrospun nanofibers [3].

Electrospinning has widely been used in biomedical engineering, including wound dressings, filtration, and drug delivery systems, as well as tissue engineering scaffolds [4]. Electrospinning process depends on several parameters, including [5] the properties of solution (viscosity, elasticity, electrical conductivity, and surface tension), applied voltage, nozzle–collector distance, ejection speed, surrounding temperature, humidity, air flow rate, etc. Therefore, the precise control of each parameter directly affects the morphology of the nanofibers [6]. In vivo, tissue engineering scaffolds must be not only a three-dimensional structure which is required to mimic extracellular matrix but also a high porosity, large surface area, suitable pore size, and highly interconnected pore structure [7]. Therefore, it is challenging for the traditional electrospinning method to obtain such biocompatibility, biodegradability, non-toxicity, and structural integrity scaffolds precisely due to the randomly intertwined nanofibers [8, 9]. Hence, the urgent requirement on electrospinning is how to precisely control the morphology and diameter of electrospinning so that it can produce thinner nanofibers with the structure of three-dimensional in biomedical engineering.

As an emerging technology, robotics has involved in and benefits many biofabrication process to ensure and improve the accuracy, flexibility, and controllability, such as 3D printing, 3D plotting, nanoimprinting. Robot-aided electrospinning is integrating robot to general electrospinning process to improve the control of parameters, the diameter of nanofibers, the rate of producing nanofibers, and so on. In this review, we summarize the state of the art of electrospinning in biomedical engineering and discuss how the robotics benefit the electrospinning process, i.e., including its working principle, main applications, challenges, and prospects.

## Basic principle and technique of electrospinning

### Basic principle

As the one of the most straightforward and cost-effective method, electrospinning technique with unique physiochemical property has gained an extensive application in biomedical field [10–12]. An integrated electrospinning device consists of a DC high-voltage supply, a syringe is filled with polymer solution, a needle, and a collector. To fabricate nanofibers, one electrode of DC high-voltage supply is connected to the needle of the syringe, and the polymer is ejected to the target collector from the top of the needle. During the process, the polymer droplets are held by the surface tension at the needle, which collects the charge on the surface induced by the electric field, while it receives an electric field force which is opposite to the surface tension [13]. The droplets are pulled from spherical to cone-shaped structure named Taylorcone. However, the electric field force will overcome the surface tension of the liquid when it increases to a critical value [14]. The polymer jet occurs under the influence of high electric field, resulting in extremely high-frequency irregular spiral motion [13]. Ultimately, a fiber of nanometer diameter is formed and scattered on the collector in a random manner to form a non-woven fabric [15].

### Melt electrospinning and solution electrospinning

**Fig. 1:**
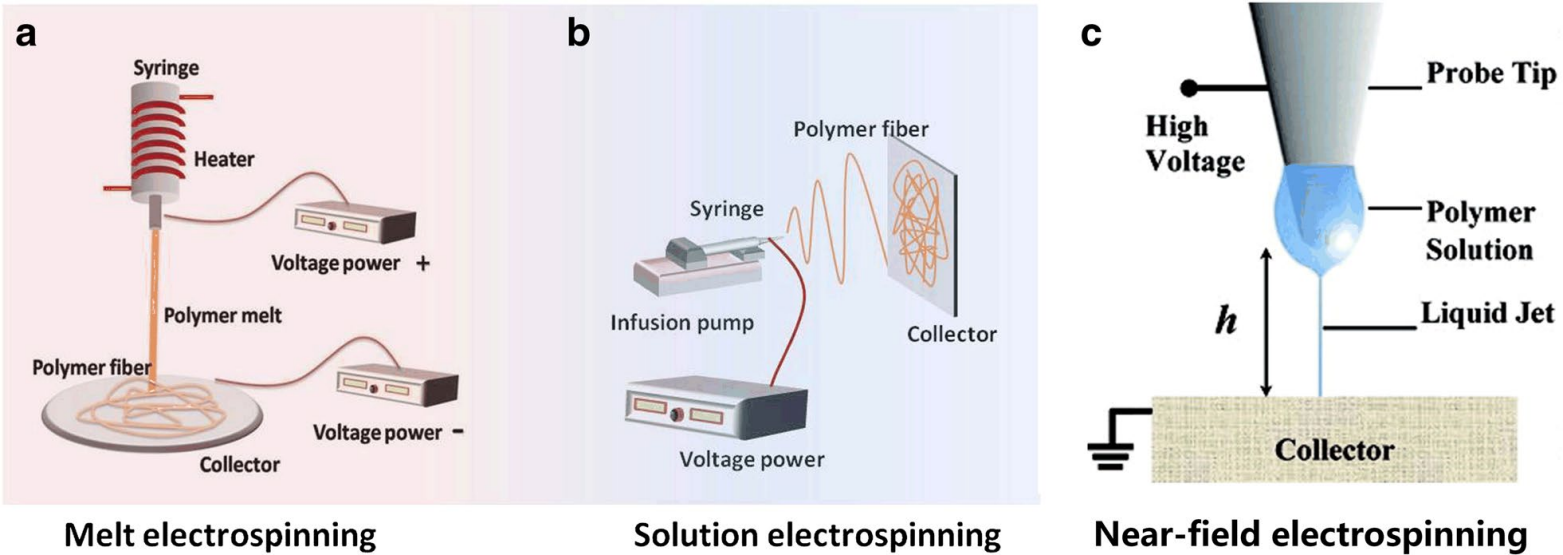
Difference in structure between melt electrospinning, solution electrospinning, and near-field electrospinning. **a** Melt electrospinning [20]. **b** Solution electrospinning [20]. **c** Near-field electrospinning [22]

The solution electrospinning is a method for the preparation of nanofibers by solvent evaporation and polymer curing under the action of high-voltage electric field, and the melt solution refers to the polymer heating and melting, by the electric field force to be drawn to obtain polymer fiber material process [16]. Structurally, both of them have a nearly identical composition, except that the melt electrospinning has an extra heater and that the solution electrospinning has an infusion pump (Fig. 1a, b). In addition, the low cost makes them a common advantage, but these two methods differentiate themselves by their typical properties.

Solution electrospinning is well known for its simplicity of operation and suitability for many polymers. The jet of solution electrospinning has the virtue of low viscosity and is easy to obtain nanofibers with diameter less than 100 nm. Besides, the surface of fiber presents the porous structure due to solvent evaporation [17]. The advantage of melt electrospinning is that there is no need to find the solvent for dissolving polymer, and the end-product is suitable for the application of biomedical engineering such as tissue engineering and drug release. In a sense, it is easy to realize the mass production due to not require the use of volatile solvents [18, 19]. While comparing these two approaches, solution electrospinning is facing the challenges of significant solvent evaporation, difficult direct-writing, and low output. In these and several respects, as a kind of raw materials with wide applicability, favorable direct-writing capability, non-toxic pollution, and high conversion rate of product technology, the melt electrospinning handles better than the solution electrospinning, but it requires severe external conditions like higher spinning temperature, more time to build, and heat-resistant polymers [20].

Nevertheless, these two techniques are difficult to achieve the requirement of high-precision pattern and structure when the polymer steps into the instability motion and splitting process [21] since the high voltage (≤ 10 kV) limits softness of nanofibers and choice of polymer materials. Finally, this could lead to create randomly coiled fibers and form uncontrolled construction. The next, the solution of spinning issues caused by the high voltage is presented.

### Near-field electrospinning

Near-field electrospinning is a technology for depositing solid nanofibers in a direct, continuous, and controllable manner by reducing the spinning distance to decrease the voltage [22]. The process is done by replacing a common needle with a tungsten needle and dipping some polymer to from droplet for spinning [22, 24]. In order to accurately control and straightly write the physical properties and precisely present 2D and 3D structure, near-field electrospinning as though jumped out in front of researchers [23, 25]. It becomes one of the most potentially technique in all sorts of fields like tissue engineering, drug delivery, biomedical engineering [26]. Compared with melt electrospinning and solution electrospinning, near-field electrospinning is a similar assembly device including high-voltage supply, probe tip, and collector (Fig. 1c), but it can achieve low-voltage electrospinning (≤ 0.2 kV) by changing conditions, direct-writing orderly and patterned nanofibers and preventing it from fabricated chaotic nanofibers [27, 28]. By comparing process, the comparison of details (Table 1) is shown by He et al. [23]. Among of all changing condition’s methods for reducing voltage, dropping off the spinning distance is regarded as the most effective way, and then adding additional conditions (e.g., magnetic field force) to decrease the electric filed also was reported by Yang et al. [29].

**Table 1:** Comparison between SES/MES and NFSE [23]

Type	Voltage (kV)	Distance (cm)	Fiber diameter (μm)	Advantages	Disadvantages
Melt electrospinning and solution electrospinning	10–30	5–50	0.01–1	Simple device Large-scale fabrication Various applicable materials	Random deposition High voltage
Near-field electrospinning	0.2–12	0.05–5	0.05–30	Controllable deposition Low-voltage precise fabrication	Immature mechanism Larger diameter Smaller-scale fabrication

Although near-field electrospinning is termed a best tool to deposit solid nanofibers in using direct-write, it is not capable enough to fabricate mass production with its single nozzle; moreover, it is easy to cause fiber diameters become thicker because of shortening spinneret-to-substrate distance [14]. Facing this a series of questions, researchers are using robot-aided for realizing large-scale production of electrospinning, and thinner nanofibers will be applied in much more fields.

## Applications of electrospinning in biomedical engineering

The main function of electrospinning technology is to prepare polymer nanofibers, which can be then designed to biomedical material, nanosensor, and nanofiber templates. As the nanofiber mats prepared by electrospinning has the characteristics of high surface-to-volume ratio, high porosity, and relatively uniform fiber diameter, it has a unique property in its application. Also, the electrospinning mats has a favorable bionic property including high biocompatibility. Then, this thesis illustrates the application in three fields.

### Applications for drug delivery

**Fig. 2:**
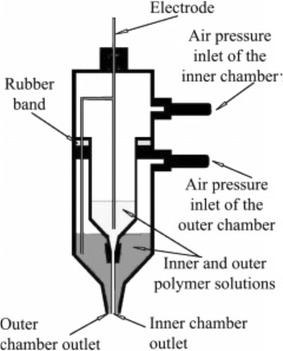
Structure of co-electrospinning or coaxial electrospinning two different polymer solutions for producing core–shell nanofibers [30]

Drug delivery systems (DDS) gained much attention in recent years, as drug-loaded materials and nanofibers mats prepared by electrospinning have many advantages, such as controlled release of drugs, little influence on the activity of drugs, and good biocompatibility. Kenawy et al. introduced that the release rates from the polyurethane, polyurethane, and their blend are similar. However, mixture of these two materials improved its visual mechanical properties [31]. Therefore, further confirmed nanofiber mats provide a scaffold with suitable mechanical strength for drug release. In terms of  drug release, Zamani et al. [32] and Jing et al. [33] have proposed some examples to achieve slow release of metronidazole by progressive degradation of PCL and using degradable PLLA as support material respectively. In addition, the core–shell structure nanofibers and multilayered drug-loaded biodegradable nanofiber can be used as a carrier material to avoid the burst release of drugs. Sun et al. [30] developed that core–shell nanofibers could be produced by co-electrospinning or coaxial electrospinning two different polymer solutions, such as polysulfone (PSU) and poly (ethylene oxide) (PEO), as well as PEO–PEO, through a spinneret comprising two coaxial capillaries (Fig. 2). On this basis, Jiang et al. [34] prepared the core–shell nanofibers by using coaxial spinning method, and controlled release of biological reagents (bovine serum and lysozyme) was realized.

On this aspect of using multilayered drug-loaded biodegradable nanofiber, Tatsuya et al. [35] developed the use of multilayered drug-loaded biodegradable nanofiber as a drug carrier material, by controlling the diameter of the fiber and the thickness of non-drug layer to achieve the purpose of controlled release. Meanwhile, making use of nanofibers prepared with the corresponding material as a drug carrier material can also achieve this aim.

Although a wide variety of drugs for the treatment of diseases have been successfully encapsulated into these nanofibers, significant progress has been made in the use of electrospun fibers for drug delivery, and many problems remain to be resolved. First, in order to produce uniform nanofibers with favorable morphological, mechanical and chemical properties for realizing its repeated and massive production are the challenge. Second, how to make drug-loading content properly and efficiently and removal of residual organic solvent are particularly important [36]. Third, we need to know that nanofibers may cause an immune response or toxicity when applying these nanofibers in vivo.

### Applications for tissue engineering

**Fig. 3:**
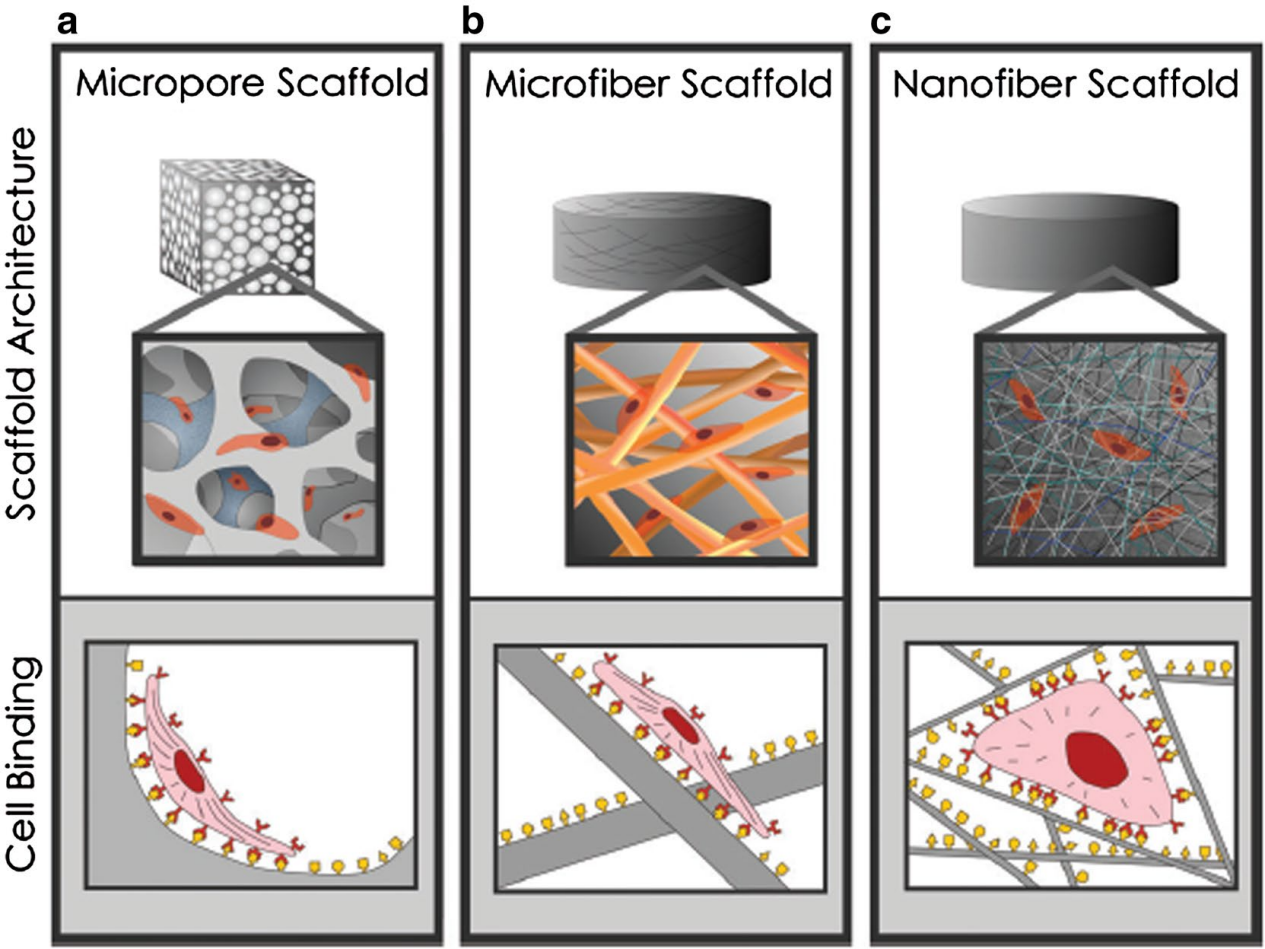
Three types of scaffold and the condition of cell binding in each scaffold. **a** Micropore scaffold. **b** Microfiber scaffold. **c** Nanofiber Scaffold [43]

Tissue engineering is an emerging discipline based on the theory of biology that utilized innovations of biology, medicine, and technology for restoring and maintaining the functions of tissues and organs [37]. The research of biomaterials plays an important role in tissue engineering by acting as substrates for the cells growth, proliferation, and new tissue formation in three dimensions [38]. In this case, the electrospinning is an low-budget, versatile, and powerful tool to produce nano- and ultrathin fibers as mimetic scaffolds to the extracellular matrix components [16]. Scaffolds with special physical characteristics like high surface-to-volume ratio are fabricated by electrospinning technique for soft tissue engineering applications in biomaterials field [39, 40], and these invention is regarded as the most conspicuous focus. Another reason for becoming the spotlight is that it has the capability to mimic the architecture of natural human extracellular matrix [40, 41], and it affects cell binding and spreading. C.T. Laurencin suggested that cells can attach and organize properly around fibers with diameters smaller than the corresponding cells [42]. Molly M. Stevens and Julian H. George reported the scaffolds with nanoscale architectures have bigger surface area for absorbing proteins by comparing type of three scaffolds (Fig. 3) [43], and the proteins further can provide an edge over microscale architectures for tissue generation applications [44]. Therefore, cells can attach and organize properly around fibers in scaffolds with nanoscale architectures.

For these reasons, nanofibrous scaffolds with its unique advantages in suitable porosity, nanoscale topography, and interconnectivity, it attracts more and more researchers constantly. About this study of nanofibrous scaffolds that better recapitulate tissue properties and enhance regeneration [7], numerous research works have been done and a lot of optimum design methods were presented. A variety of polymeric nanofibers have been considered for use as scaffolds for engineering tissues like skin tissue engineering [45, 46], bones [47, 48], vessels [49, 50], heart [51, 52], etc. In 2011, Szentivanyi et al. developed a cost-efficient and versatile approach to generate three-dimensional scaffolds of different shape and size [53]. Basu et al. succeed in researching and producing PEO and CMC/PEO nanofibrous scaffolds with 3D porous network using electrospinning technique. In addition, it is shown experimentally that nanofibrous scaffolds have thermally stable characters and the appreciable tensile properties for cell adherence and growth. MTT experimental results show that nanofibrous scaffolds possess the property of non-toxicity and cell proliferation [54].

However, there are several challenges that need to be solved prior to use of electrospun grafts in clinical applications. On the aspects of improving nanofibrous scaffolds, we should consider some important parameters such as fiber formation, morphology, composition, as well as homogenous cell distributions. Accurate bionic scaffold is the goal that researchers have been hoping to achieve.

Besides, the lack of cellular infiltration continues to be the key of research with new techniques developed to solve this challenge including dropping fiber packing density, multilayered electrospinning, dynamic cell culture, and cell electrospraying [7].

### Applications for wound dressing

The electrospun non-woven mat was fabricated by electrospinning which is a unique and versatile technique. It possesses lots of advantages such as high air permeability, high liquid absorption rate, and the flexible fitness to the wound site [55, 56]. In addition, electrospun non-woven was regarded as material to be used in wounding-healing process [57], because it can keep the wounds dry and prevent them from infection [58]. Melaiye et al. [59] developed electrospun nanofibers as carrier and loaded-silver imidazole ring composite, and their anti-bacterial action was studied for wound-healing materials. Hong and Kyung Hwa reported a PVA/Ag composite nanofiber mats as wound repair materials, due to the bactericidal effect of Ag nanoparticles, and it is possible to prevent wound infection and promote wound healing [60]. Wei et al. introduced the non-woven wound dressing with core–shell structured fibers, which was prepared by coaxial electrospinning and then taking silver nanoparticles (Ag-NPs) into the shell, whereas the vitamin A palmitate (VA), healing-promoting drug, was encapsulated in the core.

**Fig. 4:**
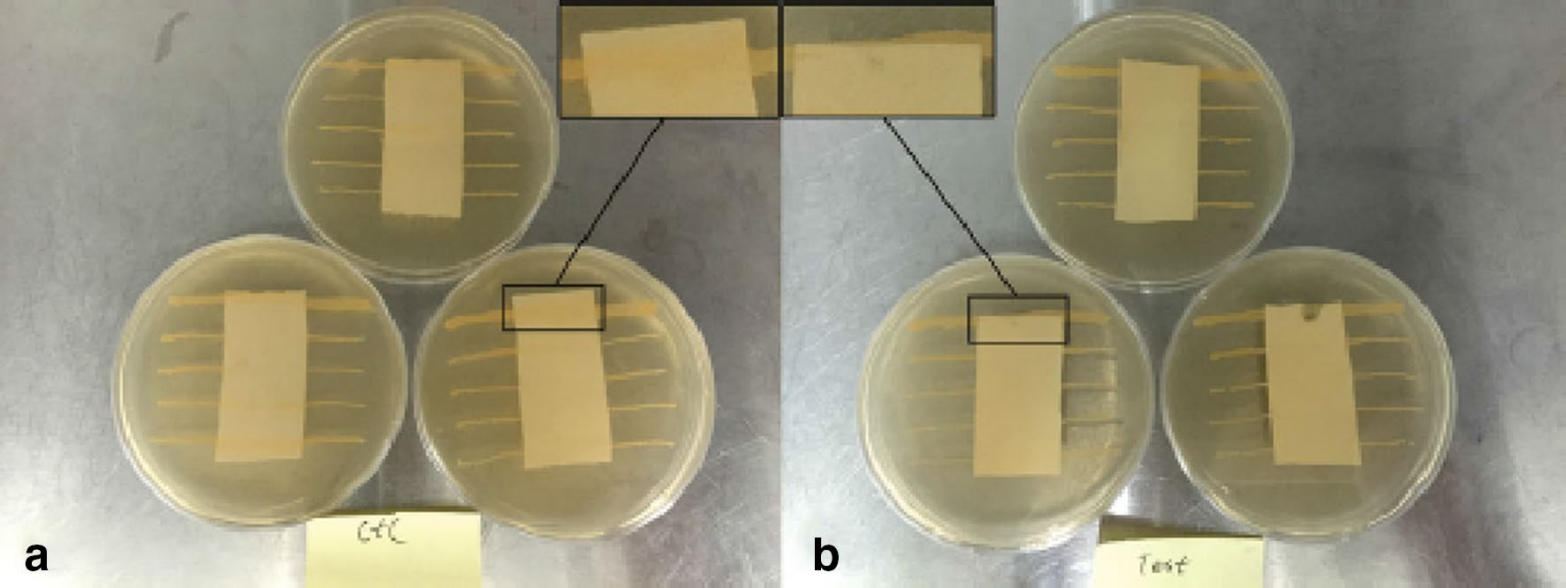
Anti-bacteria evaluation against *S. aureus*: **a** dressings (no adding); **b** dressings loaded with Ag-NPs and VA [61]

Furthermore, the dressing’s anti-bacterial ability against *Staphylococcus aureus* was proved by in vitro anti-bacterial test. The result shows (Fig. 4) *Staphylococcus* was not inhibited in the medium without loading with Ag–NPs and VA (Fig. 4a). By contrast, (Fig. 4b) shows a significant inhibitory effect with the disappeared bacteria line. This illustrates that this non-woven wound dressing has the capacity to be used as clinical wound-healing dressing [61].

However, the need of this non-woven wound dressing for all trauma is not ideal because the skin wound is heterogeneous between the patient groups, and thus the skin regeneration should turn to personalized treatment [62]. In addition, the possibility of its residual solvent is so high, and it is difficult to produce a uniform nanofiber. But it is easy to overcome by adjusting the parameters [63].

## Robot-aided electrospinning

As mentioned above, traditional electrospinning has its merits as well as its limitations and demerits. Firstly, it is difficult for the traditional electrospinning to accurately control the direction of electrospinning and get the specific three-dimensional structure in experiment. Secondly, it is difficult to apply the nanofiber mats in medical engineering directly due to the roughness of the produced nanofiber surface. Beyond that, it also difficult to achieve high productivity due to the individual nozzle. In order to realize the position-controlled deposition and precise integration of individual or aligned fibers with flexible and functional devices [23], robotics attracted the interest of researchers and attention of the electrospinning field. Robotics provides better ability to move the needle flexibly on the *x*–*y*–*z* axis because of direct computer system control. Under the control of the processing, the precise three-dimensional structure presented in front of the researchers. In addition, robotics induces the change of each parameter in the spinning process from the computer system and further controls the process of electrospinning for keeping the morphology and diameter of nanofibers.

### Robot-aided multi-nozzle electrospinning

To improve the efficiency for mass production, researchers proposed robotics multi-nozzle electrospinning technique, which makes it possible to increase the productivity and covering area [64]. In this process, the accuracy is highly affected by the repulsion from the adjacent jets and the non-uniform electric field on Taylorcone of every needle. Around these problems, a number of robot-aided multi-nozzle NFEC apparatus were presented. Many researchers hope to utilize increase the number of nozzle to achieve the robot-aided control of uniform electric field.

**Fig. 5:**
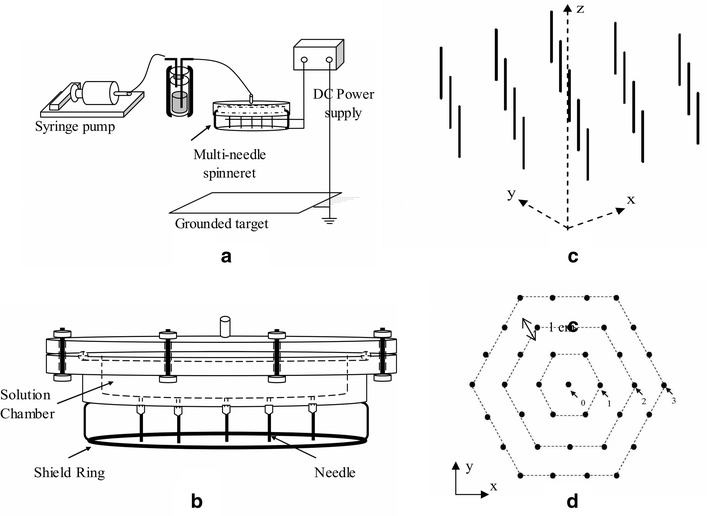
Robotics multi-nozzle electrospinning was designed by Yang et al. [65]. **a** The overall structure of the multi-nozzle electrospinning. **b** The details of the spinneret with a shield ring. **c**, **d** In *x*–*y*–*z* or *x*–*y* plane, the 3D and 2D needle distributions were presented

In order to realize a robot-aided uniform electric field strength, Yang et al. [65] utilized the design of an equilateral triangle with each set of three needles and used a shield ring to increase equilateral hexagon distributed. Based on this, Fig. 5a, b shows the basic theory and the specific construction method of Yang’s design, summarizing the feasibility of bringing a shield ring to form robot-aided uniform electric field and subsequently uniform fibers at a high production rate from two dimensions and three dimensions (Fig. 5c, d).

**Fig. 6:**
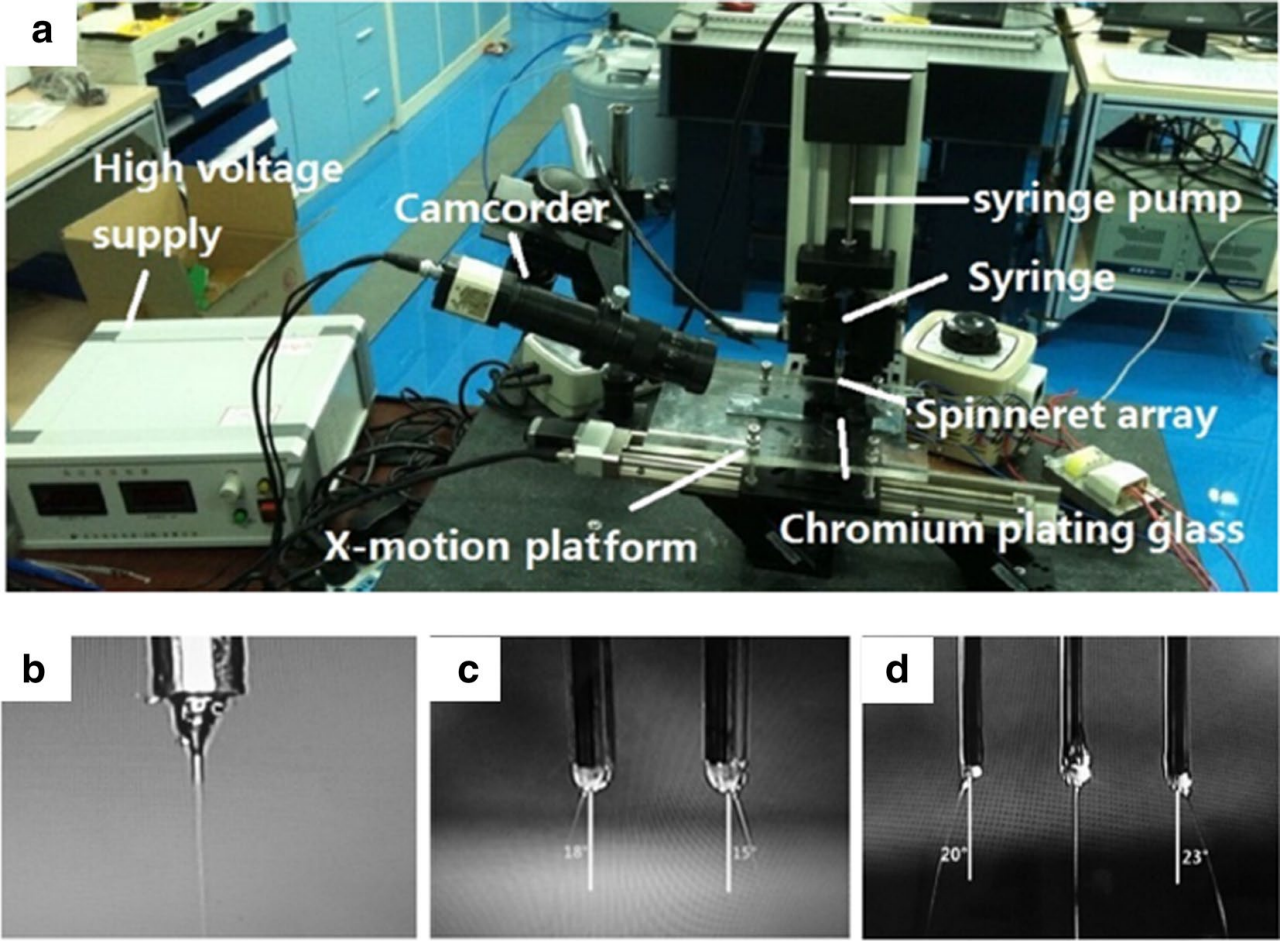
Robotics multi-nozzle electrospinning was designed by Wang et al. [66]. **a** The overall structure of the multi-nozzle electrospinning. **b** Single-nozzle NFES. **c** Double-nozzle NFES. **d** Triple-nozzle NFES

In 2015, Wang et al. [66] developed a robot-aided multi-nozzle NFEC (with double-nozzle NFES and triple-nozzle NFES) for reflecting the effect of the non-uniform electric field generated at the nozzle tip (Fig. 6a). These structures is made of high-voltage supply, syringe, X-motion platform, chromium-plated glass and camcorder, the X-motion platform, and chromium-plated glass form the collector, and syringe consists of syringe pump and spinneret array; the high-voltage supply was connected to the spinneret array, which provided a constant voltage.

**Fig. 7:**
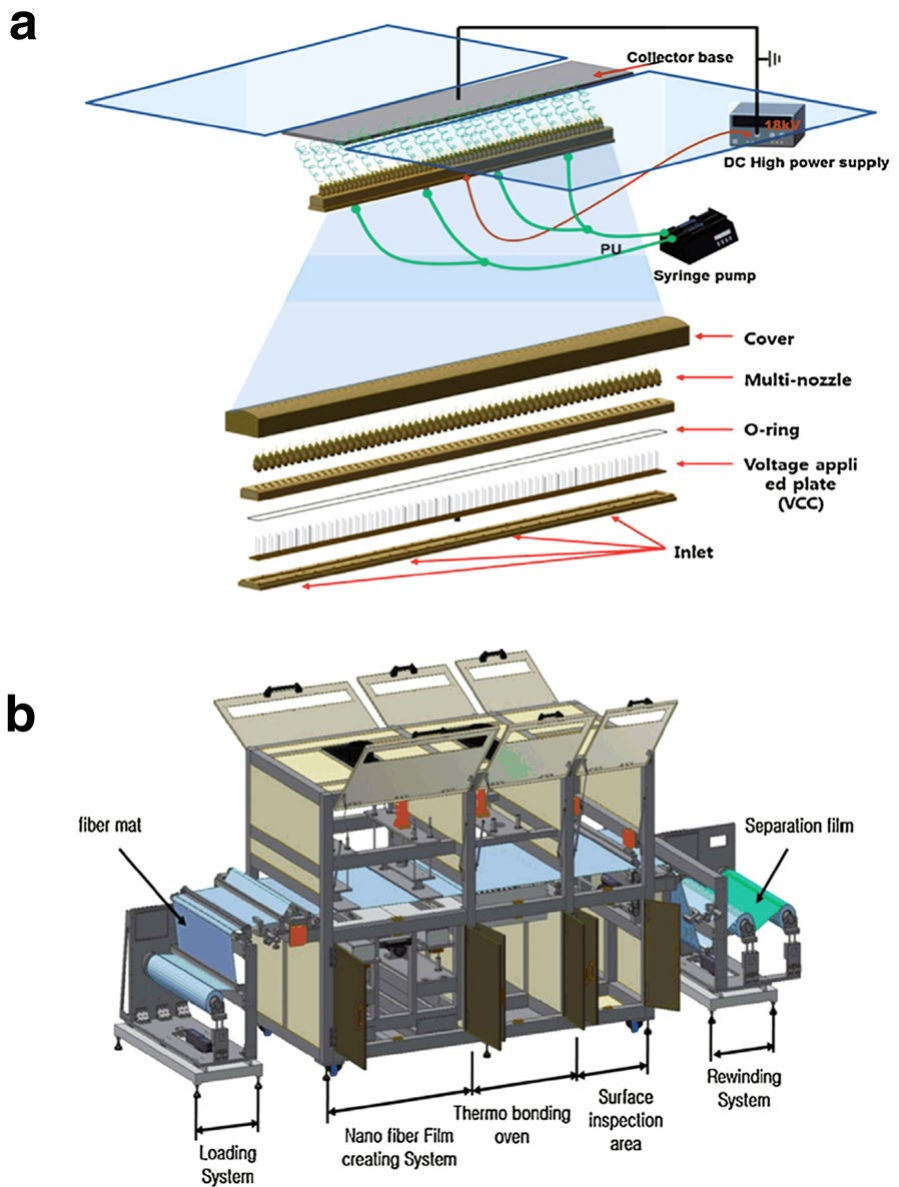
Large-scale production for fabricating nanofibers by Kim et al. [67]. **a** The overall structure of the multi-nozzle electrospinning system. **b** The nanofiber mass production system

During the process, a camcorder was used to observe the morphological variation of NFES jets which showed from single nozzle to triple nozzle (Fig. 6b–d). It should be clear that the jets could not keep the vertical line in process of between double-nozzle NFES and triple-nozzle NFES, respectively. Experimental results show the mutual distance of deposition was mainly affected by nozzle spacing and working distance rather than the voltage, and furthermore the Coulomb force is one of the major causes of interference for these phenomena, and it will lay the foundation for the further study. Meanwhile, Kim et al. [67]. developed 1-m cylinder-type high-speed robot-aided multi-nozzle setup which have 120 Ea multi-nozzles installed into cylinder block module (Fig. 7) to overcome above-mentioned problems for realizing the large-scale production and commercial operation. As a further mass production technique, researchers saw possibilities in it. However, by increasing the number of nozzle sense, a series of technological advances were not large enough to give a suitable field distribution for fabricating thinner or three-dimensional nanofibers. Therefore, researchers need to develop a robotically controlled movable multi-nozzle setup.

### A robot-assisted angled multi-nozzle electrospinning device

**Fig. 8:**
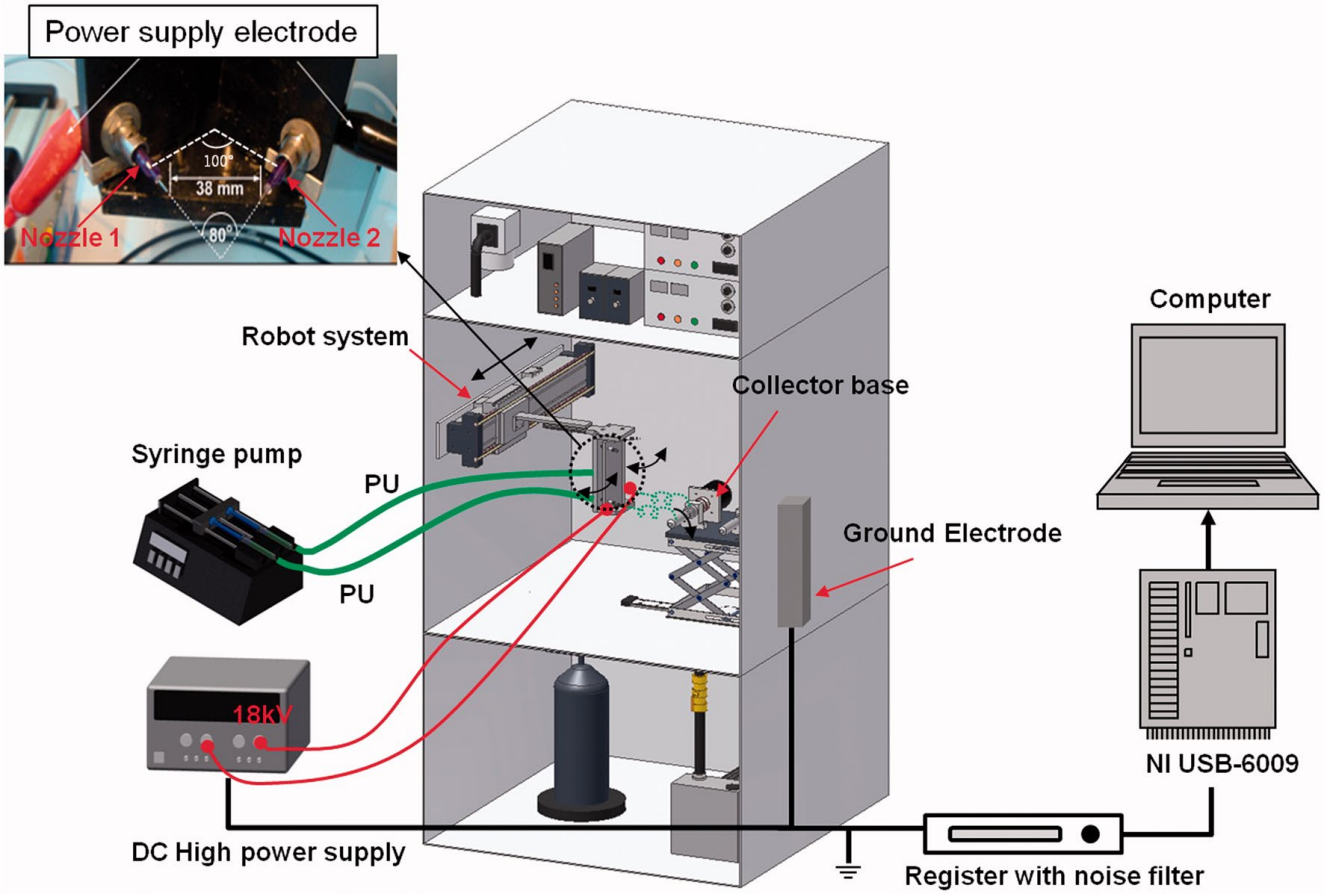
Robot-assisted angled multi-nozzle electrospinning setup by Park et al. [73]

**Fig. 9:**
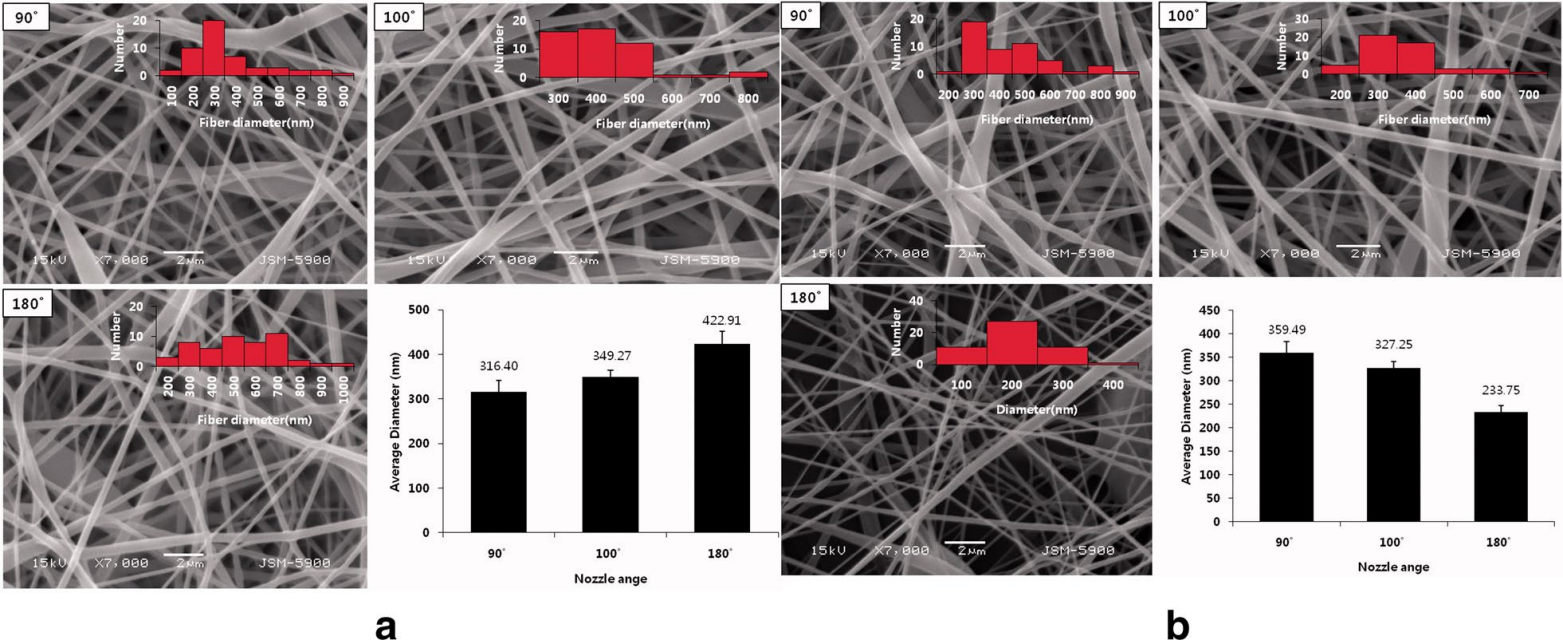
SEM images and fiber diameter distribution of electrospun fibers at different nozzle configurations by Park et al. [73]. **a** When the nozzle holder was fixable. **b** When the nozzle holder was movable

During the process of electrospinning, different parameters have been reported to change the physicochemical properties of fibers [68, 69]. As the core of estimation, parameters affect the uniform and morphology of fibers [70, 71]. Hence, to obtain uniform and hybrid material fibers by comparing different parameters, Park et al. [72] developed a multi-nozzle electrospinning setup with tunable angle between the tips of the nozzles (Fig. 8). It was composed of a removable and easy-to-control automated robot system controlled by the LabVIEW 9.0 program, two separate high-voltage power supplies created a connection to the two metal capillary nozzles, respectively, a cylindrical collector with Teflon sheet, two 10-ml plastic syringes, and two syringe pumps [72, 73]. The moveable nozzle can be easily controlled via a fully automated robot-aided system, and Fig. 6a shows the morphology and diameter of different electrospun mats when changing the angle of configurations (90°, 100°, and 180°) with nozzle holder held in the fixed position. The diameter of fibers increases with the number of angle configurations. By contrast, the diameter reduces along with the angle configurations increasing (Fig. 9b) when the nozzle holder was allowed to move sideways (horizontally, back and forth) on its axis. The results indicated that the thickness of nanofibers could be flexible operated by adding robot-aided system [73, 74].

### A robot electrospinning direct-clothing device

**Fig. 10:**
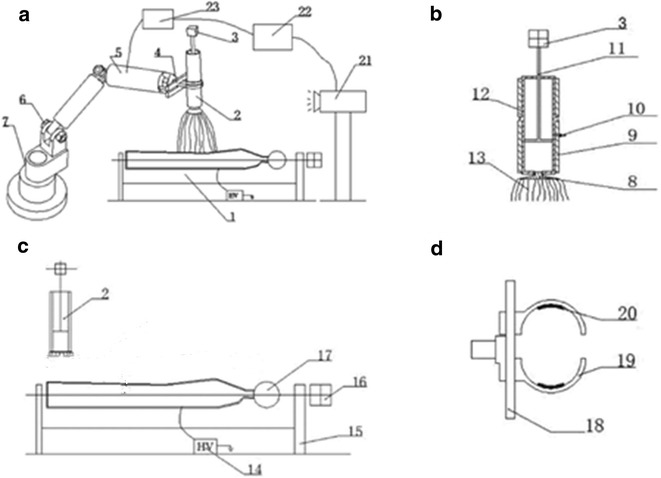
Robot electrospinning direct-clothing device by Yang et al. [75]. *a1* A robot controller, *a2,* *c2* a electrostatic spinning device, *a3*, *b3* a spinning machine, *a4* the end effector, *a5*, *a6* the robot joint, *a7* a robot, *b8* nozzle, *b9* heating ring, *b10* temperature sensor, *b11* piston, *b12* charging barrel, *b13* the jet of electrospinning, *c14* electrostatic generator, *c15* holder, *c16* model motor, *c17* a spinning model device, *d18* clamp, *d19* finger clamp, *c20* pressure sensor, *a21* a tracking camera, *a22* a computer control device, *a23* a robot electrospinning device

As mentioned above, the feasibility of producing strong plasticity and thick nanofibers with mass production is analyzed based on the principle of robot-assisted angled multi-nozzle electrospinning. In 2013, Yang et al. [75] developed a robot electrospinning device which could be achieved in the true sense of large-scale production and apply it on garment industry. An electrospinning robot (Fig. 10a23), a robot controller (Fig. 10a1), a electrostatic spinning device (Fig. 10a2), a spinning model device (Fig. 10c17), a tracking camera (Fig. 10a21), and a computer control device (Fig. 10a22) altogether consist of the robot electrospinning direct-clothing device (Fig. 10).

These systems could combine robot technology and multi-needle electrospinning and is suitable for using on solution, melt, near-filed electrospinning, and composite electrospinning. The spinning device can be operated along a predetermined path by means of a electrospinning robot induction, feedback, and control combined with a computer program control, and ensures spinning sprinkler head is vertically downward. The computer control device is mainly be responsible for carrying out data processing and conversion according to the information taken and transmitted by the tracking camera, controlling the robot controller at the same time, and the robot controller accepts the command, directs, and controls the electric spinning robot to use the electrospinning device to complete the command indication. According to the transfer of modified information feedback, the shape of electrospun model and the spinning path are modified timely. Therefore, it overcomes the disadvantage that nanofibers cannot be plasticized and operated flexibly.

### A rotating robot multi-nozzle electrospinning device

**Fig. 11:**
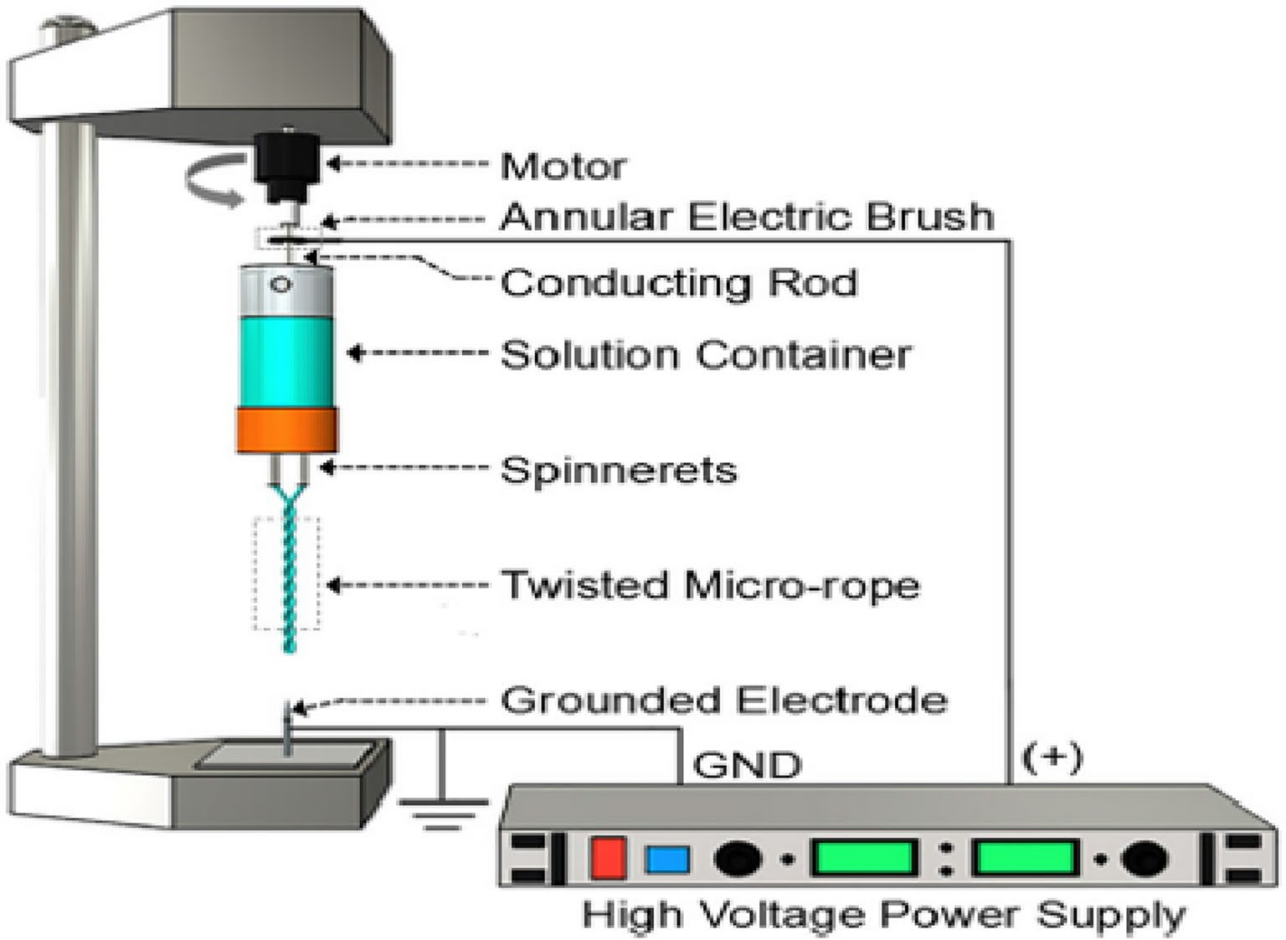
Rotating robot multi-nozzle electrospinning device by Zhang et al. [76]

Different to the two robot-aided ways mentioned above, a rotating robot multi-nozzle was developed by controlling fiber intercalation and the number of fibers involved in the overall multi-fiber structure for displaying better mechanical characteristics [77–79] and getting the preferred morphological choice for the textile industries [80]. In 2016, Zhang et al. [76] reported a rotating robot multi-nozzle for utilizing to fabricate continuous ropes and collecting two intercalated fibers micron-scaled rope fabrication via control on the diameter and number of twists (per length). Figure 11 shows that two spinnerets were driven by rotating motor; the two types of jet formed the twisted micro-rope in uniform electric field. They found the using of a rotating robot multi-nozzle directly impacts the deposition of twisty nanofibers. It overcomes the undesirable characteristics of nanofibers, such as poor mechanical strength, low surface roughness, and resulting structures which are randomly orientated [81].

## Conclusion and outlook

Robot-aided electrospinning provides much higher operation precision and stability, which makes the 3D construction of nanofibers possible and allows us to design the nanofiber’s shape according to the actual requirements. Yet, there are still many challenges that need to be addressed to make the truly intelligent biomedical engineering. Firstly, in the electrospinning process, the electrical field is one of the most parameters, which directly determines the fabrication accuracy. However, the current robot-aided electrospinning is mainly focusing the position control, and few is mentioned the dynamic model of the electrical field. More theoretical works in electrical field would be very helpful to establish the model of the electrospinning field. Secondly, the sensing method to monitor the condition of the fabricated fiber is still limited. To control the fabrication process precisely, one important thing is to estimate the product dynamically. However, in current electrospinning system, the only thing can do is to image the structure of the fibers. Other import information, such as the dimeter and mechanical property, has to be investigated after fabrication. Such off-line sensing technique brings big challenges to the real-time robot control. Hence, sensing fusion techniques that are able to get more information of the biofibers in real time could promote the system a lot. Lastly, an effective control strategy for the fabrication process should also be considered. As discussed above, the fabrication process is affected by many factors, such as voltage, nozzle–collector distance, solution, temperature. Considering those factors are coupled together, we shall build a model and design a strategy to adjust those parameters dynamically to achieve the desired result. Unfortunately, up to now, rare related works have been done, and the mechanism behind is still not clear. Therefore, more effort should be taken in this direction.

In summary, the robot-aided electrospinning is an emerging highly interdisciplinary field, which required both the knowledge of robotics and biomedical. In the future, a deep integration of the general electrospinning and the robotic should be a way to address the existing challenges. From the perspective of device design, controllable design extends the concept of “thinner” design, aiming at developing products to apply the various biomedical fields. It is particularly important to develop a simple and high accuracy of robot-aided electrospinning for saving time, simple assembling, disassembling, and maintaining.
